# The Directional Solidification, Microstructural Characterization and Deformation Behavior of β-Solidifying TiAl Alloy

**DOI:** 10.3390/ma12081203

**Published:** 2019-04-12

**Authors:** Ning Cui, Qianqian Wu, Jin Wang, Binjiang Lv, Fantao Kong

**Affiliations:** 1 School of Mechanical and Automotive Engineering, Qingdao University of Technology, Qingdao 266520, China; wuqq2012@126.com (Q.W.); jinwangqtech@163.com (J.W.); lbj@qut.edu.cn (B.L.); 2 State Key Laboratory of Advanced Welding and Joining, Harbin Institute of Technology, Harbin 150001, China; kft@hit.edu.cn

**Keywords:** TiAl, deformation, directional solidification, lamellar microstructure

## Abstract

A β-solidifying Ti–43Al–2Cr–2Mn–0.2Y alloy was directionally solidified by the optical floating zone melting method. The microstructure is mainly characterized by γ/α_2_ lamellae with specific orientations, which exhibits straight boundaries. The β phase is randomly distributed in the lamellar microstructure, indicating that the β phase cannot be directionally solidified. The directional solidification of γ/α_2_ lamellae was not affected by the precipitation of the β phase. Hot compression tests show that the deformation behavior of the β-containing lamellar microstructure also exhibits the anisotropic characteristic. The deformation resistance of the lamellae is lowest when the loading axis is aligned 45° to the lamellar interface. Microstructural observation shows that the decomposition of the lamellar microstructure tends to begin around the β phase, which benefits from the promotion of a soft β phase in the deformation. Moreover, the deformation mechanism of the lamellar microstructure was also studied. The bulging of the γ phase boundaries, the decomposition of α_2_ lamellae and the disappearance of γ/γ interfaces were considered as the main coarsening mechanisms of the lamellar microstructure.

## 1. Introduction

γ-TiAl alloys are known as promising candidates for Ti-based superalloys in aerospace industries because of their low density, high strength and good creep resistance [1,2,3]. High-performance Ti-based alloys have been used extensively for aeronautic and biomedical applications, due to their outstanding combination of properties [4,5,6,7]. By contrast, the density of γ-TiAl alloys is only about half that of Ti alloys, which is more beneficial for the weight-reduction of advanced equipment. However, low room temperature ductility and poor hot workability limit the engineering applications of TiAl alloys [8]. Many studies have confirmed that microstructure refinement and better hot workability can be achieved by introducing the β phase [9,10]. Thermomechanical treatments can be used for β-containing TiAl alloys to further refine the microstructure and improve mechanical properties [11,12]. Thus, the so-called β-solidifying TiAl alloy has attracted special attention in recent years [13]. 

The lamellar microstructure is a typical feature of TiAl alloys. The mechanical behavior of TiAl alloys are directly relevant to the lamellar orientation [14]. Fully lamellar microstructures generally exhibit greater high-temperature strength and fracture toughness than duplex microstructures [15]. TiAl alloys with an aligned lamellar microstructure have optimum tensile properties when the lamellar boundary is parallel to the loading [16]. Obviously, it is meaningful to clarify the relationship between the mechanical properties and the lamellar orientation of TiAl alloys. Directional solidification (DS) technology is an effective way to control the lamellar microstructure in TiAl-based alloys, which is conducive to this research. Previous research mainly focused on conventional (γ + α_2_) two-phase TiAl alloys. The directional solidification and mechanical properties of (γ + α_2_) TiAl alloys have been well studied. However, limited research has been conducted on the directional solidification of β-solidifying TiAl alloys, and has been mainly focused on the creep properties of β-solidifying γ-TiAl alloys. Compared with conventional TiAl alloys, the β-solidifying TiAl alloy contains α, γ and β phases. The effect of the β phase on the directional solidification of the lamellar microstructure is unclear. Moreover, the deformation resistance and deformation behavior of lamellae would also be influenced by the introduction of the β phase. Thus, further studies are needed to clarify these problems mentioned above.

In this paper, a directionally solidified β-solidifying Ti–43Al–2Cr–2Mn–0.2Y alloy was produced by optical floating zone melting. The morphology and distribution of the β phase were observed. Moreover, hot compression tests were carried out, and the hot deformation behavior, microstructural evolution and deformation mechanisms of the β-containing lamellae were investigated.

## 2. Experimental

A Ti–43Al–2Cr–2Mn–0.2Y ingot (Φ110 × 250 mm) was fabricated by vacuum induction melting. X-ray fluorescence (XRF, PANalytical, Almelo, Overijssel, Netherlands) spectrometry revealed that the actual composition of the ingot was Ti–43.4Al–1.9Cr–2.05Mn–0.18Y. A master batch (Φ6 × 100 mm) was cut from the ingot and mechanically polished to a 0.05 μm finish. A directionally solidified TiAl bar (Φ6 × 100 mm) was then prepared by the optical floating zone melting method at a growth rate of 20 mm•h^−1^ under high-purity argon. XRF showed that the actual composition of the bar was Ti–43.2Al–1.8Cr–2.1Mn–0.19Y. The dimension of the sample employed for the compression test was Φ4 × 6 mm. Hot compression specimens were electro-discharge machined parallel to the lamellar boundary of the middle columnar crystal. All compression specimens were polished using 2000 grit emery paper. Isothermal compression tests were conducted using a Gleeble-1500D simulator (DSI, Saint Paul, MN, USA) at different temperatures with a constant strain rate of 0.01 s^−1^. The phase composition was checked using X-ray diffraction (XRD, Panalytical, Almelo, Overijssel, Netherlands). Microstructural observation was conducted by scanning electron microscopy (SEM, FEI, Hillsboro, OR, USA) in the back-scattered electron (BSE) mode. In order to study the microstructural evolution and deformation behavior of the lamellar microstructure, transmission electron microscopy (TEM, FEI, Hillsboro, OR, USA) was also employed. TEM foils were prepared through mechanical polishing and twin-jet electropolishing by using a solution of 6% perchloric acid + 34% butanol + 60% methanol at −20 °C and 25 V.

## 3. Results and Discussion

### 3.1. Initial As-Cast Microstructure of β-Solidifying TiAl Alloy

**Figure 1:**
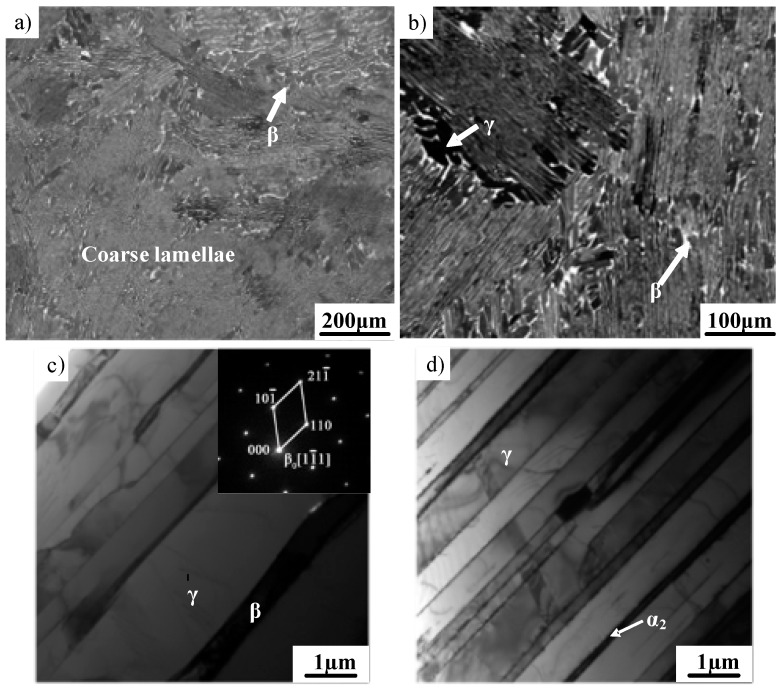
As-cast microstructure of the Ti–43Al–2Cr–2Mn–0.2Y ingot. (**a**,**b**) Coarse lamellar colonies (SEM), (**c**) β phase (TEM), (**d**) γ/α_2_ lamellae (TEM).

Figure 1 shows the as-cast microstructure of the Ti–43Al–2Cr–2Mn–0.2Y ingot. As shown in Figure 1a,b, the initial microstructure is mainly characterized by randomly oriented coarse γ/α_2_ lamellae. The average lamellar colony size is in the range of 200–600 μm. Moreover, the β phase was introduced by adding 2% Cr and 2% Mn. The XRD pattern of as-cast Ti–43Al–2Cr–2Mn–0.2Y alloy has been reported in the literature [17]. It should be noted that the β phase is a disordered phase at high temperatures, but exists as an ordered β_0_ phase with a B2 structure (CsCl) at room temperature. For convenience of expression, both disordered β and ordered β_0_ are denoted by β in this paper. It can be seen in Figure 1b,c that an irregular β phase with bright contrast and a γ phase with black contrast were distributed along the lamellar colony boundaries. A block-shaped γ phase generally precipitated around the β phase because a high-temperature β phase would transform into γ and ordered β_0_ phases during cooling [18]. The TEM image in Figure 1d shows that the initial γ/α_2_ lamellae exhibit relatively straight interfaces.

### 3.2. Directionally Solidified Microstructure of β-Solidifying TiAl Alloy

**Figure 2:**
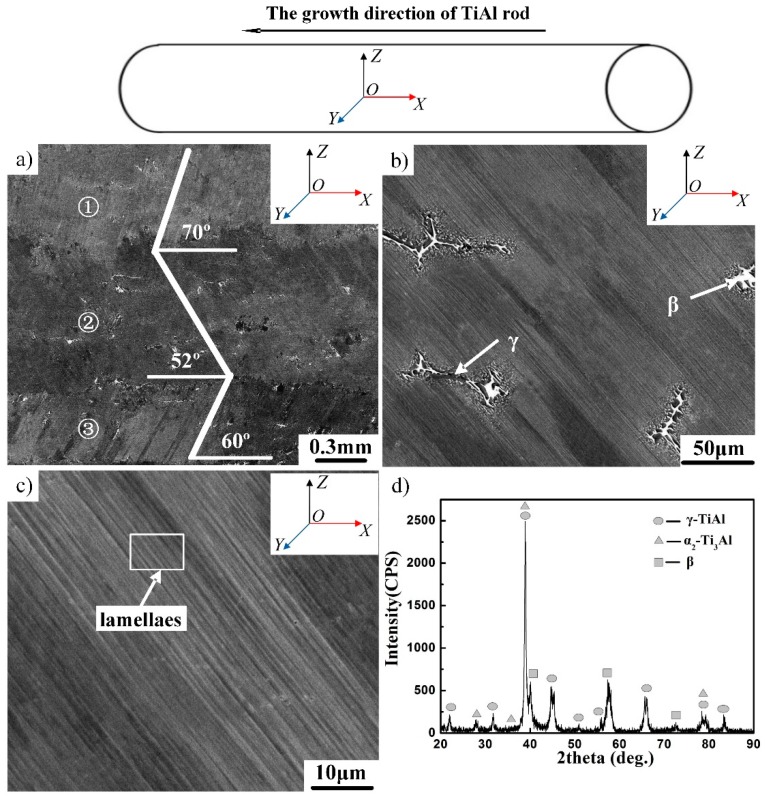
Microstructure and X-ray diffraction (XRD) pattern of directionally solidified Ti–43Al–2Cr–2Mn–0.2Y alloy. (**a**) Columnar crystal, (**b**) the distribution of β and γ phase, (**c**) lamellae, (**d**) XRD pattern.

The microstructure of the directionally solidified alloy is shown in Figure 2. As shown in Figure 2a, three columnar crystals can be observed clearly. Each columnar crystal is characterized by a lamellar microstructure with a specific orientation. Lamellar orientations of three columnar crystals were aligned 72°, 52° and 60° to the growth direction. The lamellar orientation depends on the primary phase. Based on previous studies, the values of the theoretical angle θ between the lamellar orientation and the growth direction should be 0°, 45° and 90° [19]. This is different from the actual observation in the present study, which can be ascribed to the cutting position. For θ = 0° or 90°, the observed angle would not be affected by the cutting position. Thus, the actual angle θ of the present alloy is 45°. As shown in Figure 2b, a β phase with a bright contrast was randomly distributed in lamellae, indicating that the β phase cannot be directionally solidified. The directional solidification of γ/α_2_ lamellae was not influenced by the β phase. Moreover, it can also be observed that a block-shaped γ phase precipitated around the β phase in the directionally solidified microstructure, which is consistent with the as-cast alloy [20]. As shown in Figure 2d, the phase composition was also checked using XRD and it was confirmed that the phase identity is similar to the local structure obtained from SEM and TEM.

**Figure 3:**
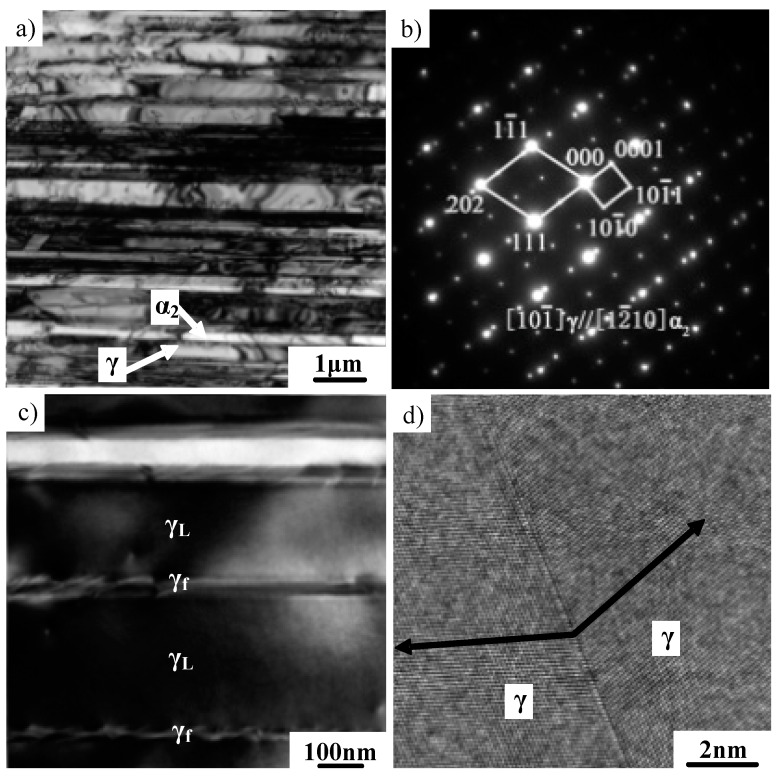
TEM images showing the microstructure of Ti–43Al–2Cr–2Mn–0.2Y alloy with a specific lamellar orientation. (**a**) γ/α_2_ lamellae, (**b**) selected area electron diffraction (SAED) patterns of γ/α_2_ lamellae, (**c**) γ/γ lamellae, (**d**) high-resolution image of the γ/γ interface.

TEM observation was also conducted to investigate the directionally solidified microstructure. The bright-field image in Figure 3a shows that the lamellar microstructure consists of fine γ and α_2_ lamellae, which exhibit straight boundaries. The selected area electron diffraction (SAED) patterns from α_2_ + γ colonies in Figure 3b identified that the γ and α_2_ phases still obey the Blackburn orientation relationship, which is consistent with the conventional TiAl alloy [21]. Moreover, plenty of γ/γ lamellae can be observed in the lamellar microstructure, as shown in Figure 3c. The γ_f_ domain embedded in the γ_L_ lamellar structure was often observed. Detailed research on γ_f_ and γ_L_ has been reported in the literature [22]. The high-resolution TEM in Figure 3d revealed that the most frequently observed γ/γ interface exhibits a twin relationship. It should be noted that Figure 3c,d are referring to two different positions on the sample.

### 3.3. Deformation Resistance of β-Containing Lamellae

**Figure 4:**
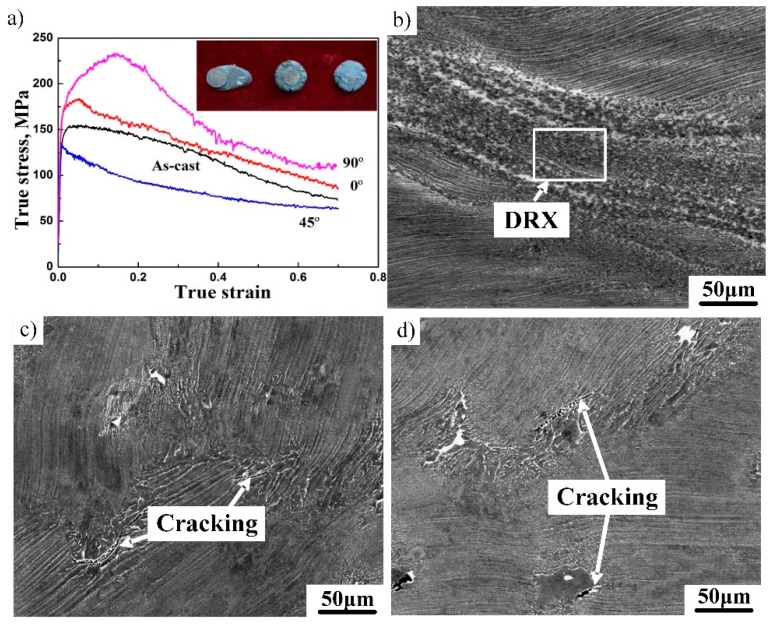
The effect of lamellar orientation on the deformation resistance and microstructural evolution of Ti–43Al–2Cr–2Mn–0.2Y alloy. (**a**) True stress–true strain curves, (**b**) 45°, (**c**) 0°, (**d**) 90°.

To study the effect of the lamellar orientation on the deformation resistance of β-containing lamellae, hot compression tests were conducted at 1100 °C/0.01 s^−1^. The values of the angle η between the loading axis and the lamellar interface were set to 0°, 45° and 90°. Fully dynamic recrystallization (DRX) tends to occur for the lamellar microstructure when the deformation temperature reaches 1200 °C [23]. Thus, a low test temperature (1100 °C) was adopted to observe the microstructural differences more clearly. True stress–true strain curves, compressed specimens and deformed microstructures of the alloy deformed at different conditions are presented in Figure 4. As shown in Figure 4a, all curves exhibit typical dynamic softening features. When η = 45°, the peak stress is lowest (135 MPa), which is lower than that of the as-cast alloy. It can be seen from the appearance of the deformed specimen that wedge cracking occurred, which is related to the ${\left\{111\right\}}_{\gamma }//\left(0001\right)\alpha_{2}$ orientation relationship between the γ and α_2_ phases. Shear deformation occurs more easily on the (111) close-packed plane parallel to γ/α_2_ lamellar boundaries, which is beneficial to the plastic deformation and the decrease of the deformation resistance. Thus, DRX generally tends to occur in the shearing area, as shown in Figure 4b. In addition, it can be found that the magnitude of the β phase increased significantly after plastic deformation. This can be ascribed to the phase transformation α→β + γ during hot deformation, which was identified by Takeyama [20]. In contrast, when η = 0° or 90°, the peak stress reaches 184 MPa and 233 MPa, respectively, indicating that the deformation is difficult in both directions. The dislocation glide is hindered by the γ/α_2_ lamellar boundaries, leading to an increase in the deformation resistance. As shown in Figure 4c,d, some cracks formed due to high deformation resistance. High deformation resistance also affects the decomposition of the lamellar microstructure. The deformation behavior of the β-containing lamellar microstructure also exhibits an obvious anisotropic characteristic. A homogeneous and fine microstructure was the expected and desired microstructure, which can be obtained through severe plastic deformation. Given that β-solidifying TiAl alloy exhibits excellent hot deformability, it can be foreseen that multiple forgings may be an effective way for grain refinement, and further research related to this will be conducted in the future.

### 3.4. Microstructural Evolution of β-Containing Lamellae

**Figure 5:**
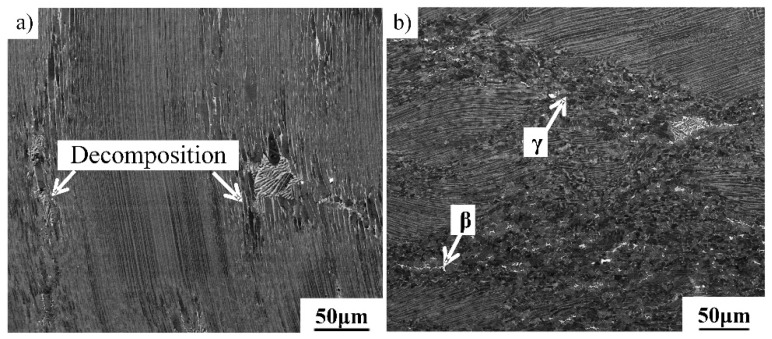
The effect of deformation magnitude on the microstructural evolution of Ti–43Al–2Cr–2Mn–0.2Y alloy. (**a**) 20%, (**b**) 40%.

To study the hot deformation behavior and the microstructural evolution of β-containing lamellae, the directionally solidified alloy was isothermally compressed with different deformations at 1200 °C/0.01 s^−1^. The stress axis is aligned parallel to the lamellar orientation of the middle columnar crystal. The microstructure of TiAl alloys after 20% and 40% hot deformation are shown in Figure 5a,b, respectively. As shown in Figure 5a, γ/α_2_ lamellae exhibit only small bending and coarsening when the deformation amount is 20%. Only a few new grains formed around the initial β phase, indicating that the decomposition of the lamellar microstructure tends to occur around the β phase. This is because a soft β phase contributes to hot deformation and promotes DRX. As can be seen from Figure 5b, a large quantity of fine grains formed when the deformation amount increased to 40%, indicating that the decomposition of the lamellar microstructure was effectively promoted by large deformation during hot working. The microstructure mainly consists of a fine equiaxed γ phase, little β phase and many residual lamellae. These phases are further confirmed in the following section. The residual lamellae are mainly due to the inhomogeneous deformation of the lamellar microstructure, as discussed in Section 3.3. The lamellae tend to decompose completely in the region where plastic deformation occurs more easily.

**Figure 6:**
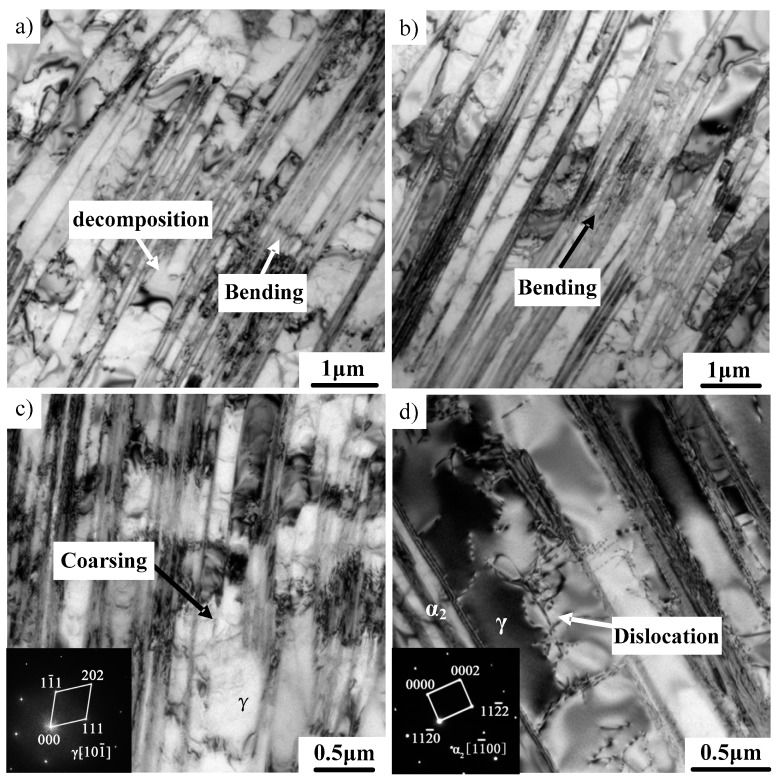
TEM images showing the microstructure of the Ti–43Al–2Cr–2Mn–0.2Y alloy after 20% deformation. (**a**,**b**) Bended lamellae, (**c**) coarsening of lamellae, (**d**) dislocations.

**Figure 7:**
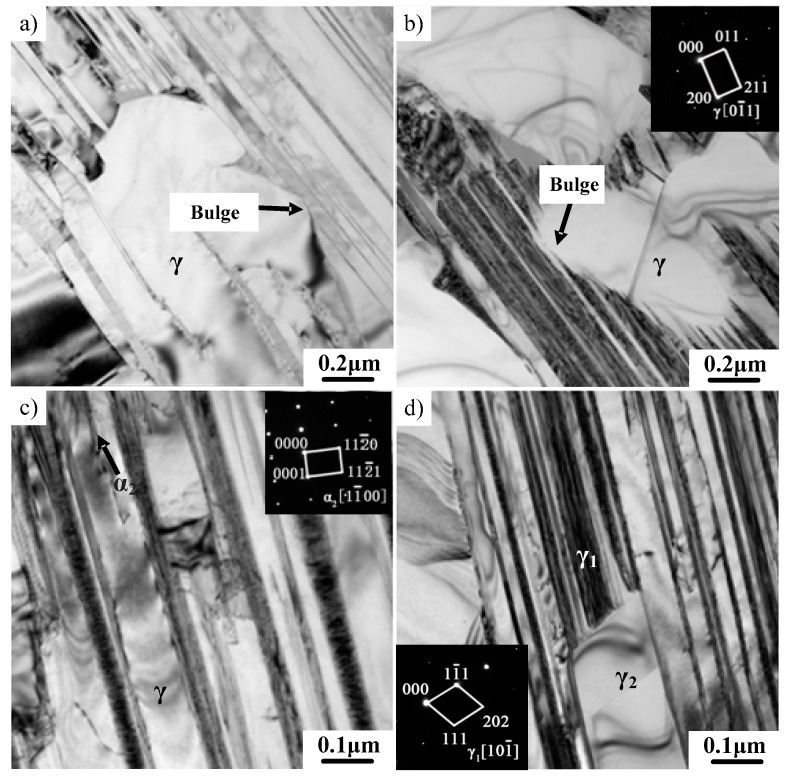
TEM images showing the coarsening mechanism of γ lamellae. (**a**,**b**) The bulging of phase boundaries, (**c**) the decomposition of α_2_ lamellae, (**d**) the decomposition of the γ phase.

Previous studies have made great progress in understanding the microstructural evolution of TiAl alloys during hot working. However, the deformation mechanism is not yet clear. Further TEM investigation was conducted on the microstructure of TiAl alloys with different deformations to study the deformation mechanism. As shown in Figure 6, bending and coarsening occurred in γ/α_2_ lamellae in the deformed microstructure (20% reduction). Compared to the initial microstructure, the uniformity of the lamellar width significantly decreased. The corresponding SAED pattern identified that the coarsening mainly occurred on γ lamellae. Several neighboring fine lamellae are easily transformed into coarse γ lamellae [24,25]. As shown in Figure 7a,b, the bulging of the γ phase boundaries are obviously observable, which is regarded as a major way to destroy lamellar boundaries. The boundaries of the γ phase propagate and destroy initial lamellar microstructure with the increase of the deformation. It can also be found that newly formed fractured lamellae possess the characteristic of sharp corners, which is caused by the bulging of the γ phase boundaries. As shown in Figure 7c, the decomposition of the α_2_ phase between γ phases also occurred, thereby merging two thin γ lamellae on either side of the α_2_ phase into new thick γ lamellae. A similar phenomenon was also observed in other deformed alloys [26,27]. The microstructure of as-cast TiAl alloy is actually at a nonequilibrium state due to a high cooling speed, which results in the formation of a metastable α_2_ phase. A metastable α_2_ phase transforms into a γ phase under high temperature and large stress, leading to the decomposition of α_2_ lamellae. Moreover, it can be seen from Figure 7d that the dissolution of fine γ lamellae resulted in the coarsening of γ lamellae. According to previous research, three types of γ lamellar interfaces were identified [28]. The true twin γ/γ interface is relatively stable. The pseudo-twin γ/γ interface and the 120° rotational fault γ/γ interface are thermally unstable. These unstable γ/γ interfaces tend to disappear by ledge migration and interface bulging during hot deformation. Therefore, the coarsening mechanisms of γ/α_2_ lamellae can be obtained, including the bulge of the γ phase boundaries, the decomposition of α_2_ lamellae and the disappearance of unstable γ/γ interfaces. 

**Figure 8:**
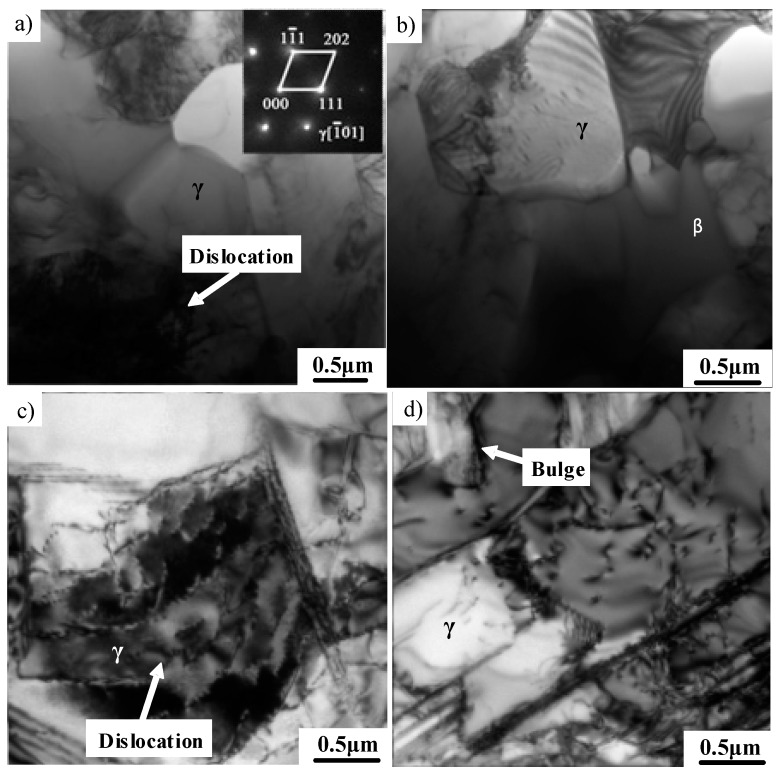
TEM images showing the microstructure of the alloy after 40% deformation. (**a**) Recrystallized γ grains, (**b**) the coexistence of the γ and β phases, (**c**) dislocations, (**d**) sub-boundaries.

TEM images of the microstructure of the TiAl alloy after 40% hot deformation are shown in Figure 8. The microstructure in Figure 8a shows that a great amount of fine equiaxed γ grains were formed, which is a typical characteristic in the large deformed region. The increased deformation remarkably accelerated the decomposition of the lamellar microstructure. High-density dislocations and sub-grain boundaries caused by the deformation enhanced the stored energy in TiAl alloys. When the deformation reached a certain degree, a recrystallized nucleus was formed in the high-density dislocation accumulation area, and then the crystal nucleus was transformed into a new recrystallized grain. Meanwhile, the dislocation density decreased significantly. The stress concentration was released. SAED patterns identified that DRX mainly occurred in the γ phase, which is ascribed to the low stacking fault energy (SFE) of a γ phase with the face-centered cubic (fcc) structure. By contrast, both the α phase with the hexagonal close-packed (hcp) structure and the β phase with the body-centered cubic (bcc) structure exhibited high SFEs. The main hot deformation mechanism for α and β is dynamic recovery. As shown in Figure 8b, some recrystallized γ grains tended to appear around the β phase. The deformation can be promoted by a soft β phase, which is beneficial to the occurrence of DRX. The morphology of residual γ lamellae was also observed. As shown in Figure 8c, the dislocation density in γ lamellae obviously increased and many dislocation substructures formed. The γ phase had more independent slip systems than the α_2_ phase, and the activation of dislocations in the α_2_ phase also required higher shear stress. Thus, the γ phase mainly affords the plastic deformation of TiAl alloys. As can be seen from Figure 8d, some low-energy sub-grain boundaries were formed by the rearrangement of dislocations as the hot deformation increased, thereby promoting the decomposition of lamellae. A similar bulging mechanism of the γ phase boundaries was also observed in Figure 8d. 

## 4. Conclusions

(1) A novel β-solidifying TiAl alloy was fabricated by directional solidification. Microstructural observation showed that the β phase was distributed randomly in the lamellae, indicating that the β phase cannot be directionally solidified. γ/α_2_ lamellae can be controlled by directional solidification, which was not influenced by the precipitation of the β phase.

(2) The deformation behavior of the β-containing lamellar microstructure also exhibited an anisotropic characteristic. When the angle η between the loading axis and the lamellar interface was 45°, the lamellae exhibited the lowest deformation resistance.

(3) The decomposition of γ/α_2_ lamellae tended to begin around the β phase. The bulging of the γ phase boundary, the decomposition of α_2_ lamellae and the disappearance of γ/γ interfaces are the main coarse mechanisms of γ lamellae.
